# lncRNA FR215775 Regulates Th2 Differentiation in Murine Allergic Rhinitis

**DOI:** 10.1155/2022/7783481

**Published:** 2022-06-14

**Authors:** Yue Ma, Le Shi, Keqing Zhao, Chunquan Zheng

**Affiliations:** ^1^Department of Otolaryngology-Head and Neck Surgery, Eye, Ear, Nose, and Throat Hospital, Fudan University, 83 Fenyang Road, Xuhui District, Shanghai 200031, China; ^2^Shanghai Key Clinical Disciplines of Otorhinolaryngology, Shanghai, China

## Abstract

To identify the effect of long noncoding RNA (lncRNA) FR215775 in regulating CD4+ T cells on murine models of allergic rhinitis (AR), the expression of lncRNA FR215775 in primary Th2 cells was detected through qRT-PCR. After knocking down the expression of lncRNA FR215775 via Sh-FR215775-Ads, Cell Counting Kit-8, cytometric bead array, and fluorescence-activated cell sorting were performed to determine its functions in vitro. Moreover, lncRNA FR215775-silencing or nonsilencing cells were injected intravenously into AR mice. Then, hematoxylin and eosin, Alcian blue-periodic acid Schiff, and toluidine blue staining were performed, and the levels of IL-2, IL-4, IL-5, IL-6, IL-10, IL-17A, IFN-*γ*, and TNF in the AR mice were also determined. We found that the expression of lncRNA FR215775 was specifically higher in the murine primary Th2 cells. After the knockdown of lncRNA FR215775, the proliferation of CD4+ T cells was inhibited, and the expressions of IL-4 and IL-5 in the cell culture supernatant were significantly decreased (*P* < 0.001), along with the percentage of Th2 cells (*P* < 0.05). The lncRNA FR215775-silencing AR group showed less serious allergic symptoms and a low level of ovalbumin-specific immunoglobulin E (*P* < 0.01). Meanwhile, the eosinophilia inflammation, goblet cell hyperplasia, and mast cell inflammation in the nasal mucosa all decreased, which indicated attenuated allergic inflammation in the lncRNA FR215775-silencing AR group. In addition, the Th2-related cytokines IL-4 and IL-5 were downregulated in the serum and nasal lavage fluid of this group (*P* < 0.01). In conclusion, lncRNA FR215775 may play a vital role in the function and differentiation of Th2 cells, which may encourage allergic inflammation. These results may provide significant insights into AR pathogenesis and offer new treatment targets for alleviating AR.

## 1. Background

Allergic rhinitis (AR) is a common and persistent allergic disease that affects the lives of many people and results in a considerable economic burden. In the past 10 years, many breakthroughs have been made in the pathogenesis of AR. Among them, imbalance of the differentiation process from primitive CD4+ T cells to Th1 and Th2 cells is one of the main factors leading to allergic reactions [1]. However, the mechanism leading to the excessive activation of Th2 cells remains unclear.

Long noncoding RNA (lncRNA) is a kind of RNA with a length of more than 200 base pairs. It has not received much attention because it has no protein-coding function. However, since the development of sequencing technology, it has been revealed that lncRNA participates in various biological processes, including cell proliferation, differentiation, and apoptosis [2, 3]. In addition, lncRNA is involved in regulating the immune response and influences the differentiation and activation of various immune cells, such as dendritic cells, B cells, and T cells [4–7]. Although various Th1- and Th2-cell-specific lncRNAs have been discovered [8, 9], their roles in the activation and differentiation of CD4+ T cells remain unclear. Furthermore, there have been few reports on the roles of lncRNAs in AR and other allergic diseases.

Our previous study detected the lncRNA profile in CD4+ T cells using the spleens of mice with AR. Bioinformatics was used to analyze related signal pathways and to construct a coexpression network of lncRNAs and mRNAs to screen important lncRNAs that could affect the differentiation of CD4+ T cells [10]. In that study, we found that lncRNA FR215775 was closely related to the function and differentiation of CD4+ T cells in AR mice. Therefore, we hypothesized that lncRNA FR215775 could play a vital role in the differentiation of Th2 cells in AR mice, which further encourages allergic inflammation. The study of the mechanism of lncRNA FR215775 may provide significant insights into AR pathogenesis and offer new treatment targets to alleviate AR.

## 2. Methods

### 2.1. Primary CD4+ T Cells and Th2 Cell Isolation

This study obtained permission from the local ethical committee of the Otolaryngology, Head and Neck Surgery, Eye, Ear, Nose, and Throat Hospital, Fudan University. Six-week-old female BALB/c mice (Shanghai Laboratory Animal Center, Shanghai, China) were used to establish an AR murine model, following the previously described experimental protocol [11]. The CD4+ T cells were selected as previously described. Briefly, to isolate CD4+ T cells from murine spleens, negative selection was conducted using Miltenyi beads and a MACS Vario magnetic cell separation apparatus (Miltenyi Biotec, Teterow, Germany) according to the manufacturer's protocol. The T cell purity was assessed using fluorescence-activated cell sorting (FACS) with anti-CD3-fluorescein isothiocyanate and anti-CD4-phycoerythrin. Primary Th2 cells were then positively selected according to the murine Th2 cell isolation kit instructions (Miltenyi Biotec, Teterow, Germany).

### 2.2. Extracting RNA and qRT-PCR

Total RNA was extracted from fresh cells using the TRIzol® reagent (Invitrogen). The RNA concentration was measured using NanoDrop ND-2000 (Thermo Fisher Scientific, Waltham, MA, USA). To validate the expression of lncRNA FR215775 in Th2 cells, qRT-PCR was conducted. The qPCR primer sequences that were used were lncRNA FR215775 forward primer (5′-3′) GGCTGGACAGATCTCGAAGG, reverse primer (5′-3′) TTTGAAGTCCGACTGCTCGT, internal control gene (GAPDH) forward primer (5′-3′) GGTTGTCTCCTGCGACTTCA, and reverse primer (5′-3′) TGGTCCAGGGTTTCTTACTCC. SYBR Premix Ex Taq™ (Takara Bio, Inc.) was used to conduct real-time PCR using the ABI PRISM 7500 Sequence Detection System (Applied Biosystems, Foster City, CA, USA). Both the target and reference genes were amplified in separate wells in triplicate. The comparative threshold cycle (2^-*ΔΔ*^CT) method was used to calculate the gene expression.

### 2.3. CD4+ T Cell Culture and Differentiation

The isolated murine primary CD4+ T cells were cultured in vitro with a complete medium (45 ml 1640 medium, 5 ml FBS, 5 *μ*l nonessential amino acid, 10 mM sodium pyruvate, 50 *μ*l 50 mM *β*-mercaptoethanol, and 0.1 mg/ml IL-2 100 *μ*l; Gibco, PeproTech) for 24 h. The spleen mononuclear granulocytes were irradiated with 3000 rad as antigen-presenting cells (APCs) and were then added to the cultured CD4+ T cells at a T : APC ratio of 1 : 10. A 400 *μ*g/ml OVA solution, IL-4 (20 ng/ml), and anti-IFN-*γ* (10 *μ*g/ml) were then added to the cocultured cells at the same time. The cells were cultured under TH2-polarizing conditions in a 37°C incubator for 4 days.

### 2.4. Knockdown of lncRNA FR215775

The m-FR215775 adenovirus interference vector with the GFP fluorescent marker was purchased from Shanghai Hanheng Company. The sense target sequences for sh-FR215775 and sh-control were sh-FR215775 (5′-GCAAGTTTCTGTGAAGTTA-3′) and sh-control (5′-TTCTCCGAACGTGTCACGTAA-3′). Native CD4+ T cells were infected with the viral supernatants in the presence of 8 *μ*g/ml polybrene (Millipore) and then cultured with a complete medium (45 ml 1640 medium, 5 ml FBS, 5 *μ*l nonessential amino acid, 10 mM sodium pyruvate, 50 *μ*l 50 mM *β*-mercaptoethanol, and 0.1 mg/ml IL-2 100 *μ*l; Gibco, PeproTech). Fluorescence could be observed after 48–72 h.

### 2.5. Lymphocyte Proliferation Test

The number of cells per well was adjusted to l × 10^6^/ml to inoculate a 96-well plate, and the plate was incubated at 37°C for 48 h. Then, a 10 *μ*l Cell Counting Kit-8 (CCK-8) (Yeasen Biotech Co.) solution was added to each well, and the 96-well plate was incubated at 37°C for 6 h. Finally, the plate was measured at 450 nm in an absorbance microplate reader (Thermo).

### 2.6. Cytometric Bead Array

Cytokine evaluation was performed using a cytometric bead array (CBA). The levels of cytokines, including IL-2, IL-4, IL-5, IL-6, IL-10, IL-17A, IFN-*γ*, and TNF, were quantified in cellular supernatants, nasal lavage fluid (NALF), and the blood serum of AR mice using CBA (BD Biosciences) according to the manufacturer's instructions.

### 2.7. Flow Cytometry

The Th1/Th2/Th17 cell ratio was detected through flow cytometry analysis using the Ms TH1/2/17 Phenotyping Kit (BD Biosciences). According to the manufacturer's protocol, 0.5 *μ*l/ml ConA was added to each well of all cultured cells, and the BD GolgiStop™ Protein Transport Inhibitor (4 *μ*l/6 ml) was also added at the same time. After stimulation for 5 h, a 1 ml precooled BD Cytofix™ buffer was added, and then the cells were incubated at room temperature for 10–20 min. Later, a 50 *μ*l BD Perm/Wash™ buffer was added to fully suspend the cells, and 20 *μ*l cocktail or negative control was also included. The mixture was left to incubate at room temperature for 30 min in the dark. Later, 1 ml 1× BD Perm/Wash™ buffer was centrifuged at room temperature to wash the cells twice. Then, the cells were suspended in a stain buffer and tested on the machine. FlowJo (TreeStar Inc.) software packages were used for the data analysis.

### 2.8. Grouping and Adoptive Transfer

**Figure 1:**
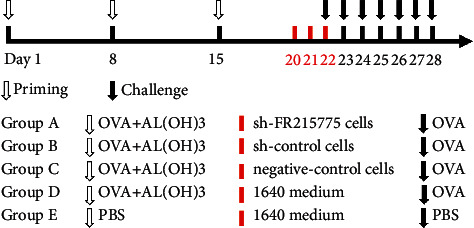
Schematic diagram of AR mice model grouping.

The mice were divided into five groups: the Sh-FR215775 intervention AR group (Group A), the Sh-control intervention AR group (Group B), the control cell intervention AR group (Group C), the nonintervention AR group (Group D), and the blank control group (Group E). Each group had five 6-week-old female SPF Balb/c mice. The mice from Groups A, B, C, and D were sensitized using intraperitoneal injection of OVA + Al (OH)3 suspension prepared with normal saline on days 1, 8, and 15 (500 *μ*g/mL OVA + 20 mg/ml Al (OH)3, 200 *μ*l/mouse). On days 20, 21, and 22, the mice in Groups A, B, and C were continuously injected intravenously with virus-transfected or control cells. Furthermore, Groups D and E were injected with the same amount of 1640 medium. From days 22 to 28, the mice from Groups A, B, C, and D continuously received intranasal excitation with OVA (40 mg/ml OVA and 20 *μ*l/mouse), and Group E received nasal drip with the same amount of PBS (Figure 1).

### 2.9. Chemical Staining

Five specimens were selected from each group. At least three random fields at ×400 magnification were selected for each specimen. HE staining was performed to observe the infiltration of eosinophils in the mouse nasal mucosa. The cytoplasm of eosinophils is rich in bright- or brick-red eosinophils. The eosinophils in the nasal mucosa were observed and counted, and the results were taken as the mean.

Alcian blue-periodic acid Schiff (AB-PAS) staining was used to observe the degree of goblet cell metaplasia and the ratio of goblet cells to epithelial cells in mouse nasal mucosa tissue. Red represents neutral glycoprotein, and blue represents acid glycoprotein. The ratio of metaplastic goblet cells in the nasal mucosa to epithelial cells was determined, and the results were taken as the mean.

In toluidine blue staining, the cytoplasm of mast cells is rich in dark-purple or purple-red particles, and the nucleus is dark blue. We thus used this stain to observe and count the number of mast cells in the tissues on both sides of the murine nasal mucosa.

### 2.10. Statistical Analysis

Statistical analysis and graphing were conducted using SPSS (version 22.0) and GraphPad Prism 7.0. Furthermore, all the data were tested for normality using the Kolmogorov–Smirnov test. The differences between the groups were analyzed using the one-way analysis of variance, *t*-test, or Mann–Whitney *U* test. *P* < 0.05 was considered statistically significant.

## 3. Results

### 3.1. Upregulation of lncRNA FR215775 Expression in AR Murine

**Figure 2:**
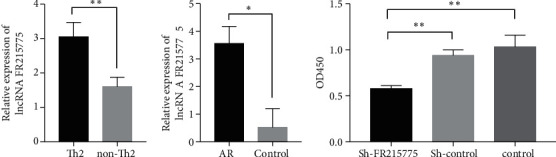
The relative expression of lncRNA FR215775 and the CD4+T cells proliferation. (a) Expression of lncRNA FR215775 was highly in primary Th2 cells and (b) in AR mice compared with the control group; (c) proliferation of cells in the FR215775 knockdown group was significantly inhibited compared with the control group (^∗^*P* < 0.05; ^∗∗^*P* < 0.01).

According to our previous research, we successfully established an AR murine model (Figure S1) and then isolated primary CD4+ T cells and Th2 cells from murine spleens. Through bioinformatics analysis, we predicted that lncRNA FR215775 was related to the T cell differentiation pathway. To further explore the role of lncRNA FR215775 in AR mice, we first examined its expression in CD4+ T cells. The qRT-PCR results demonstrated that lncRNA FR215775 was highly expressed in the primary CD4+ T cells, especially in the Th2 cells from the AR mice spleens (Figures 2(a) and 2(b)).

### 3.2. Inhibition of CD4+ T Cell Proliferation through lncRNA FR215775 Silencing

The primary CD4+ T cells were divided into three groups: the sh-FR215775 group, the sh-control group, and the control group. By continuously adjusting the virus infection concentration and the concanavalin A (ConA) stimulation concentration, we found that when the multiplicity of infection = 1,000 and ConA concentration = 5 *μ*g/ml, the virus transfection efficiency was highest, and the cell activity was optimal (Figure S1A, B). Under blue excitation light, the successfully transfected cells displayed green fluorescence (Figure S1C, D). Then, the number of green cells and the total number of cells in the same field of view under visible light irradiation were calculated separately. The transfection efficiency was about 65%. CCK-8 assay revealed that the proliferation of CD4+ T cells in the sh-FR215775 group was significantly decreased compared to the control group after 48 h of transfection (*P* < 0.01; Figure 2(c)).

### 3.3. Function and Differentiation of Th2 Cells Affected by lncRNA FR215775 Silencing

**Figure 3:**
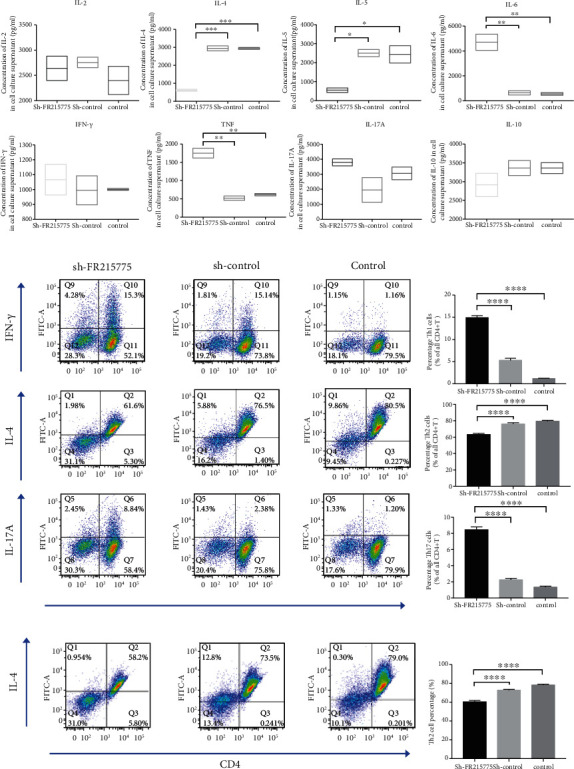
Knockdown of lncRNA FR215775 affect Th2 cell function and differentiation; (a) the concentrations of IL-4 and IL-5 in sh-FR215775 group were significantly lower than those in the control group; (b, c) the proportion of Th2 cells decreased whereas the proportion of Th1 and Th17 cells increased in the cells of sh-FR215775 group; (d, e) the proportion of induced Th2 cells was significantly reduced in the sh-FR215775 group; (^∗^*P* < 0.05, ^∗∗^*P* < 0.01, ^∗∗∗^*P* < 0.001, ^∗∗∗∗^*P* < 0.0001).

A CBA multifactor detection kit was used to detect the expression of various cytokines in the supernatant of mouse primary CD4+ T cells cultured in vitro. The results revealed that the concentrations of IL-4 and IL-5 were lower in the sh-FR215775 group than in the control group (*P* < 0.001 and 0.05, respectively). However, the secretions of IL-2, IFN-*γ*, IL-17A, and IL-10 were not significantly affected. In contrast, the expressions of IL-6 and TNF were significantly higher than those in the control group (*P* < 0.01 for both IL-6 and TNF; Figure 3(a)). Moreover, the ratios of different T cell subtypes were also detected using flow cytometry, which revealed that the proportion of Th2 cells decreased by more than 10% whereas those of Th1 and Th17 cells increased significantly in the sh-FR215775 group (Figures 3(b) and 3(c); *P* < 0.0001). These results suggest that lncRNA FR215775 is involved in the function and immune regulation of Th2 cells. In addition, after the addition of IL-4 (20 ng/ml) and anti-IFN-*γ* (10 *μ*g/ml), CD4+ T cells were polarized and induced to differentiate Th2 cells. We found that in the sh-FR215775 group, the proportion of induced Th2 cells was significantly reduced (Figures 3(d) and 3(e); *P* < 0.0001). Hence, lncRNA FR215775 may be related to the differentiation of Th2 cells.

### 3.4. Impairment of AR Murine Allergic Inflammation by lncRNA FR215775 Silencing

**Figure 4:**
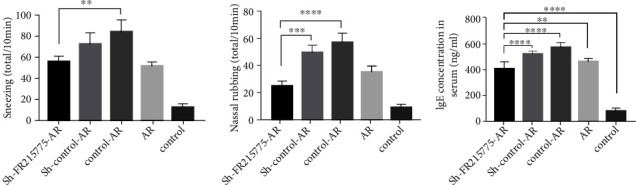
Symptoms scores and the serum IgE level in mice. (a, b) Symptoms of AR mice model can be ameliorated by lncRNA FR215775 silencing. Data represent the mean ± SD. (c) The serum IgE level was significantly reduced in sh-FR215775-AR group compared with the control AR group (^∗∗^*P* < 0.01, ^∗∗∗^*P* < 0.001, ^∗∗∗∗^*P* < 0.0001).

**Figure 5:**
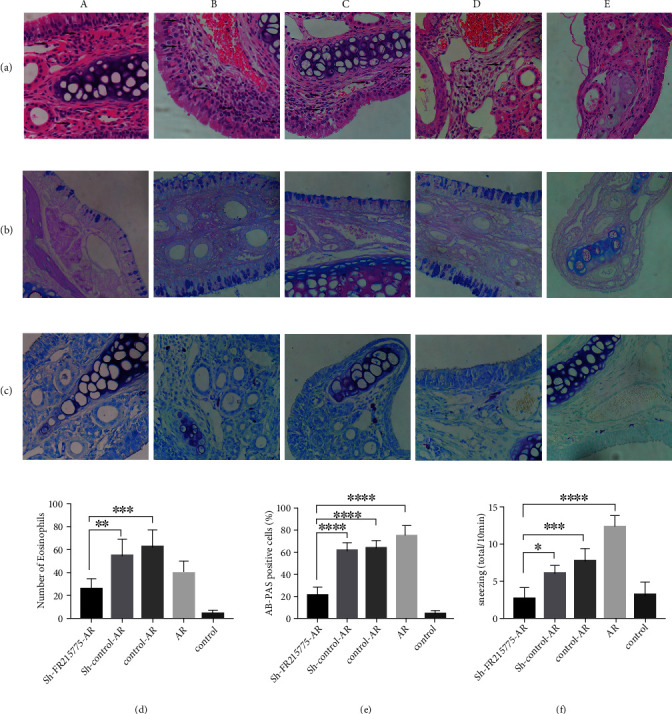
Histopathological staining of murine nasal mucosa. Tissue sections were stained with (a) HE staining, (b) AB-PAS staining, (c) toluidine blue staining to analyze the severity of allergic inflammation. Photographs of representative nasal mucosal in each group (a–c; ×400 magnification; black arrows indicate stain positive cells). (d) Number of eosinophils. (e) Percent count of goblet cells. (f) Number of mast cells was significantly decreased in sh-FR215775 group (*n* = 5 per group). (A) The sh-FR215775 intervention AR group. (B) The sh-control intervention AR group. (C) The control cell intervention AR group. (D) The nonintervention AR group. (E) The blank control group. Data represent the mean ± SD (^∗^*P* < 0.05, ^∗∗^*P* < 0.01, ^∗∗∗^*P* < 0.001, ^∗∗∗∗^*P* < 0.0001).

The sneezing and nasal scratching behaviors of Group E were slighter than those of Groups A and D. Among all these groups, Group A had a significant reduction in symptoms compared with Groups B and C, which had the same cell intervention (Figure 4(a), 4(b)). The serum IgE levels of the mice in Groups A, B, C, and D decreased significantly, but their IgE levels were still obviously higher than those of the mice in Group E (*P* < 0.01; Figure 4(c)). Large numbers of inflammatory cell infiltrations were observed in the nasal mucosae of the AR mice, including eosinophils, goblet cells, and mast cells (Figures 5(a)–5(c)). The numbers of these cells in the mucosa could reflect the degree of allergy. It was found that the number of eosinophils in Group A was significantly less than those in Groups B and C (*P* < 0.05), but there was no significant difference in this regard between the AR and nonintervention AR groups (Figure 5(d)). Statistical analysis of the percentage of AB-PAS-positive cells in the epithelial cells revealed that the goblet cells in Group A were significantly reduced and that their percentage was even lower than that in Group D (Figure 5(e)). Compared with Group E, the four other groups of AR mice all demonstrated more obvious mast cell inflammatory infiltration. Furthermore, in the nasal mucosae of the Group A mice, significant decreases in the numbers of mast cells were observed, which meant that the degree of mast cell inflammatory infiltration was reduced (Figure 5(f)).

### 3.5. Downregulation of Th2-Related Cytokines in the Sh-FR215775-Silencing Mice

**Figure 6:**
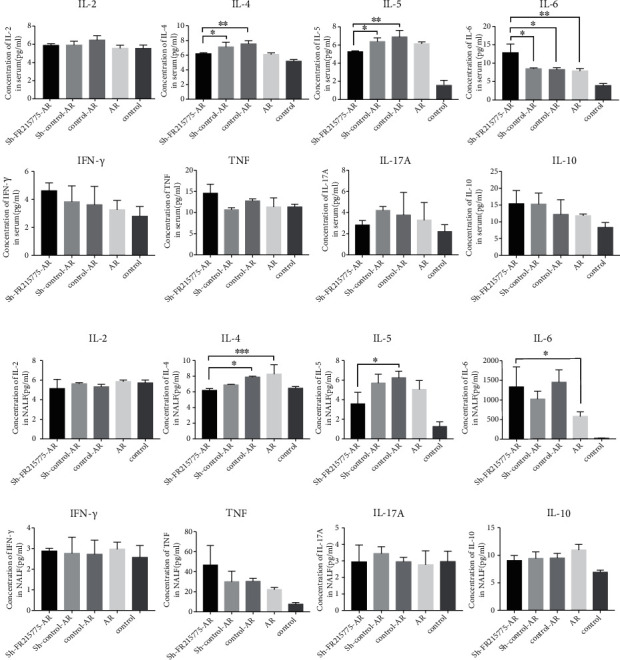
The sh-FR215775-AR group mice showed downregulation of Th2 cytokines. Expression of related cytokines in (a) murine serum and (b) murine NALF. The concentrations of IL-4 and IL-5 in lncRNA FR215775 silencing group were significantly lower than those in control group. Data represent the mean ± SD (^∗^*P* < 0.05, ^∗∗^*P* < 0.01, ^∗∗∗^*P* < 0.001).

The expression levels of IL-4 and IL-5 were lower in the serum and NALF of the mice with the intervention of virus knockdown lncRNA FR215775 cells than in those of the mice in the control group (*P* < 0.01 and 0.05, respectively). However, there was no significant difference with the AR mouse group without any intervention. In both the mouse serum and NALF, the secretions of IL-2, IFN-*γ*, IL-17A, and IL-10 were not significantly affected. In addition, the expression of IL-6 in the serum and NAFL showed an increasing trend (Figure 6). These results are consistent with the results found in vitro.

## 4. Discussion

Primary CD4+ T cells lose their balance during the differentiation process. As a result, Th2 cells are overactivated, which has always been considered the immunological basis for the pathogenesis of AR [12]. Overactivated Th2 cells produce a series of cytokines [5], which leads to higher serum IgE levels and an increase in eosinophils, mast cells, and goblet cells in the mucosal epithelium.

It has been revealed that T cells contain a variety of specific lncRNAs, and these lncRNAs have been confirmed to be involved in the differentiation, development, and activation of T cells. Many effector T cell-specific lncRNAs have been found in humans and mice, including Th1-specific lncRNA IFN-AS1 [13, 14], lnc-MAF-4 [12], Th2-specific lncRNA linc-Ccr2-5′AS [15], Th2LCRR [8], and GATA3-AS1 [16]. Moreover, lncRNA has been confirmed to play a crucial role in regulating gene expression and signal pathways, affecting the occurrence and development of various allergic diseases [17, 18] and autoimmune diseases [19], such as asthma, systemic lupus erythematosus, rheumatoid arthritis, and Crohn's disease. Furthermore, although Sarlus et al. [20] have explored gene expression in the hippocampus and anterior cortex of AR mice, there are almost no reports about research on lncRNA in T cells in AR murine models. In our previous experiments, we determined that there were large numbers of differentially expressed lncRNAs and mRNAs between AR mice and normal mice. Our results confirmed that the differential lncRNAs in the spleen CD4+ T cells of AR mice could be highly related to the pathogenesis of AR. To further explore the role of lncRNA as a specific therapeutic target in the treatment of AR, we selected several signal pathways related to T cell differentiation and constructed a coexpression network of lncRNAs and mRNAs. The results showed that the mRNA in each pathway was highly correlated with multiple differential lncRNAs. Therefore, we believe that these differentially expressed lncRNAs participate in the T cell differentiation process in AR by regulating related genes [10].

In this study, we found that the expression of lncRNA FR215775 was upregulated in the primary Th2 cells of AR murine and that knocking down the expression of lncRNA FR215775 could affect CD4+ T cell proliferation. Moreover, the expression of Th2-related cytokines was significantly decreased in the knockdown group, which further confirmed that lncRNA FR215775 could affect the function of Th2 cells. Interestingly, we found that the expression levels of IL-6 and TNF were significantly higher in the knockdown group and that the mechanism needed further exploration. In addition, knocking down lncRNA FR215775 interfered with the percentage change in each CD4+ T cell subtype. The proportion of Th2 cells decreased significantly whereas the proportions of Th1 and Th17 cells increased. These results suggest that lncRNA FR215775 can affect the function and participate in the immune regulation of Th2 cells.

Only a few studies involving murine in vivo experiments have investigated the functions of lncRNAs in T cells. Hu et al. [9] found that lincR-Ccr2-5′AS, located on chromosome 9 of mice and coexpressed with the nearby coding genes CCR1, CCR2, CCR3, and CCR5, could regulate the migration of Th2 cells to the lungs. It has been confirmed in C57BL/6 mice that the downregulated expression of lincR-Ccr2-5′AS could significantly reduce the migration of effector Th2 cells to the lungs. Therefore, future research should explore the feasibility of delaying the development of allergic diseases by interfering with the lncRNA in T cells. At present, most clinical treatments involving AR drugs act only in the final development stage of allergic effects, with short-term efficacy and long-term application of safety risks. Therefore, research on new and effective treatment methods is urgent for the treatment of AR.

In the in vivo experiments in our study, we chose to apply continuous tail vein injection to the lncRNA FR215775-silencing cells before the excitation period, hoping to affect or even block the disease process and inhibit the excitation process of the corresponding allergy mechanism in mice. Our results confirmed that knocking down lncRNA FR215775 could inhibit allergic symptoms in the nasal cavities of AR mice to a certain extent by regulating the expression levels of the related cytokines in the serum and NALF. Inflammatory cell immersion was significantly decreased in the lncRNA FR215775-silencing group compared with the negative control group. However, there were no significant differences in the various detection indicators compared to the mice in the control group (AR without cell intervention). These results revealed that lncRNA FR215775 could promote allergic reactions in the development of AR and could participate in the development of AR by regulating the Th2 cell function and the differentiation of CD4+ T cells into Th2 cells. To further confirm the allergy-promoting functions of lncRNA FR215775, further related investigations should be performed in the future. In addition, the mechanism by which lncRNA FR215775 affects the function and differentiation of CD4+ T cells is unclear. This mechanism should be further explored.

Immunotherapy through under-the-skin injections or under-the-tongue medication is the only method that provides continuous antiallergic effects after treatment discontinuation. Scadding et al. [21] have found that immunotherapy with under-the-tongue medication can reduce the levels of IL-4 and increase the inhibitors of IL-4 receptors in related patients [22, 23]. However, immunotherapy is risky and may induce severe allergic reactions in patients during the initial stages of treatment. Sastre et al. reported that a large number of pollen allergy patients experience side effects during immunotherapy [24]. Hence, a fully individualized assessment is required before the performance of immunotherapy [25], which limits its wide application. In addition, drugs for the treatment of AR mainly contain antihistamines, glucocorticoids, and hypertrophic cell stabilizers with certain side effects. We expect to inhibit the conversion of T cells to Th2 cells by interfering with Th2-cell-specific lncRNA. This will decrease the production of the corresponding cytokines, which can lead to the treatment of AR.

## 5. Conclusions

In summary, we found that lncRNA FR215775 was elevated in murine primary Th2 cells. Knocking down the expression of lncRNA FR215775 can inhibit the proliferation of murine CD4+ T cells, affecting the function and differentiation of Th2 cells. In vivo, lncRNA FR215775 deletion can impair AR murine allergic inflammation through downregulation of the level of Th2-related cytokines. However, the mechanism by which lncRNA FR215775 affects the function and differentiation of CD4+ T cells is unclear. Thus, we will further explore this mechanism to find more specific interference targets for allergic reactions and build the foundation for AR-targeted therapy. Overall, these results may provide significant insights into AR pathogenesis and offer new treatment targets for alleviating it.

## Data Availability

All data used to support the findings of this study are included within the article.

## Supplementary Materials

Supplementary 1 Establishment of AR murine model. (A) Schematic diagram of AR mice model. The number of (B) sneezes and (C) nose scratches in AR mice model was significantly increased compared to the control group. (D) The serum IgE level was significantly upregulated in AR group. (E-F) Tissue sections were stained with HE staining to analyze the severity of allergic inflammation. Photographs of representative nasal mucosal in each group. (G)Eosinophil count of each group (*n* = 5 per group). Data represent the mean ± SD (^∗∗∗^*P* < 0.001, ^∗∗∗∗^*P* < 0.0001).

Supplementary 2 The activated and virus-transfected CD4+ T cells (A) the activated CD4+ T cells became bigger after ConA stimulation. (B) Cells that emit green fluorescence under blue excitation light are successfully transfected cells (×10 objective).
