# Supplementary figures and images for: lncRNA FR215775 Regulates Th2 Differentiation in Murine Allergic Rhinitis
Source: J Immunol Res. 2022 Jun 14;2022:7783481. doi: 10.1155/2022/7783481 (PMC9214652; doi:10.1155/2022/7783481)

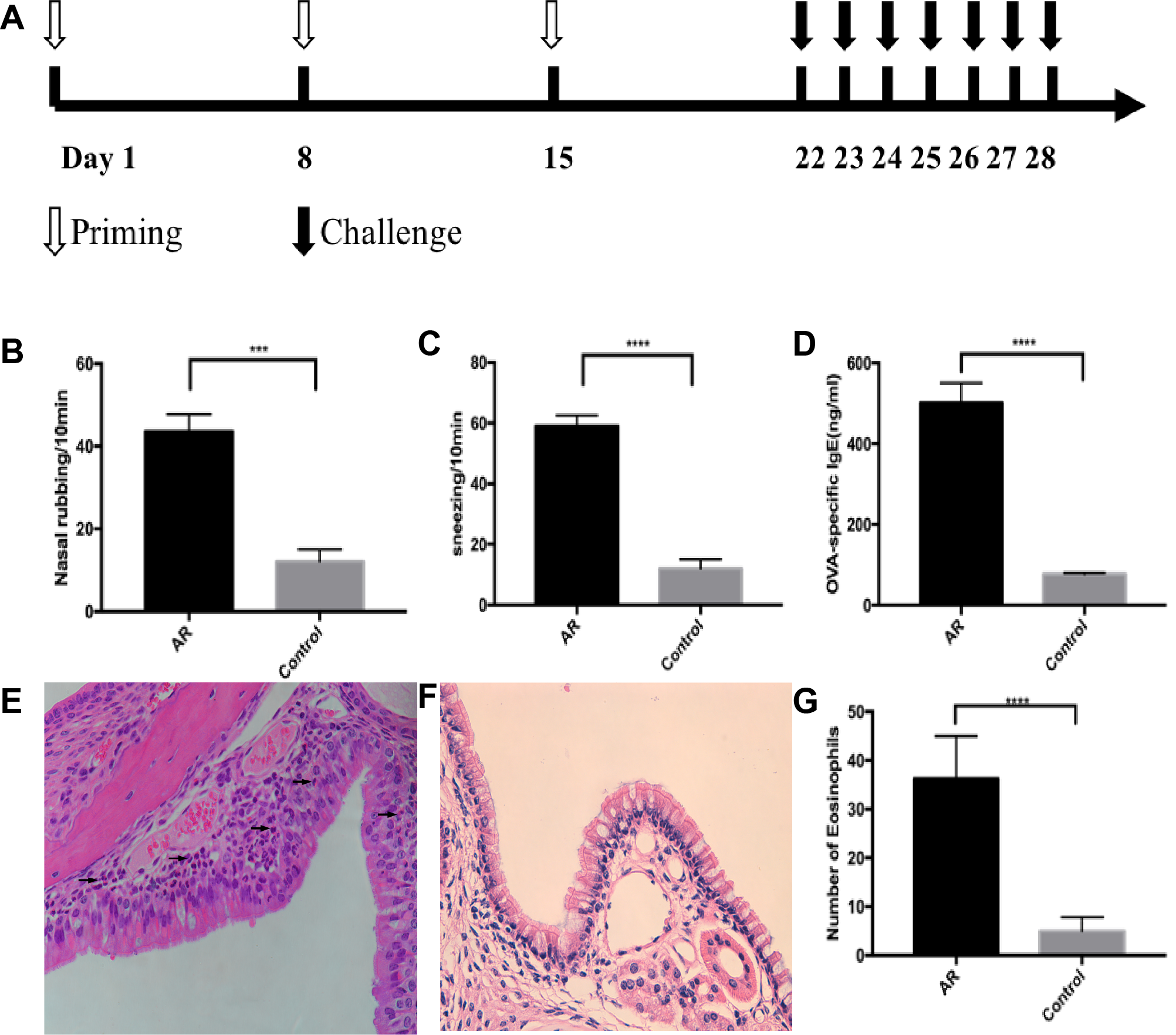

Supplement: Supplementary 1 — Establishment of AR murine model. (A) Schematic diagram of AR mice model. The number of (B) sneezes and (C) nose scratches in AR mice model was significantly increased compared to the control group. (D) The serum IgE level was significantly upregulated in AR group. (E-F) Tissue sections were stained with HE staining to analyze the severity of allergic inflammation. Photographs of representative nasal mucosal in each group. (G)Eosinophil count of each group (n = 5 per group). Data represent the mean ± SD (∗∗∗P < 0.001, ∗∗∗∗P < 0.0001). [file 7783481.f1.png]

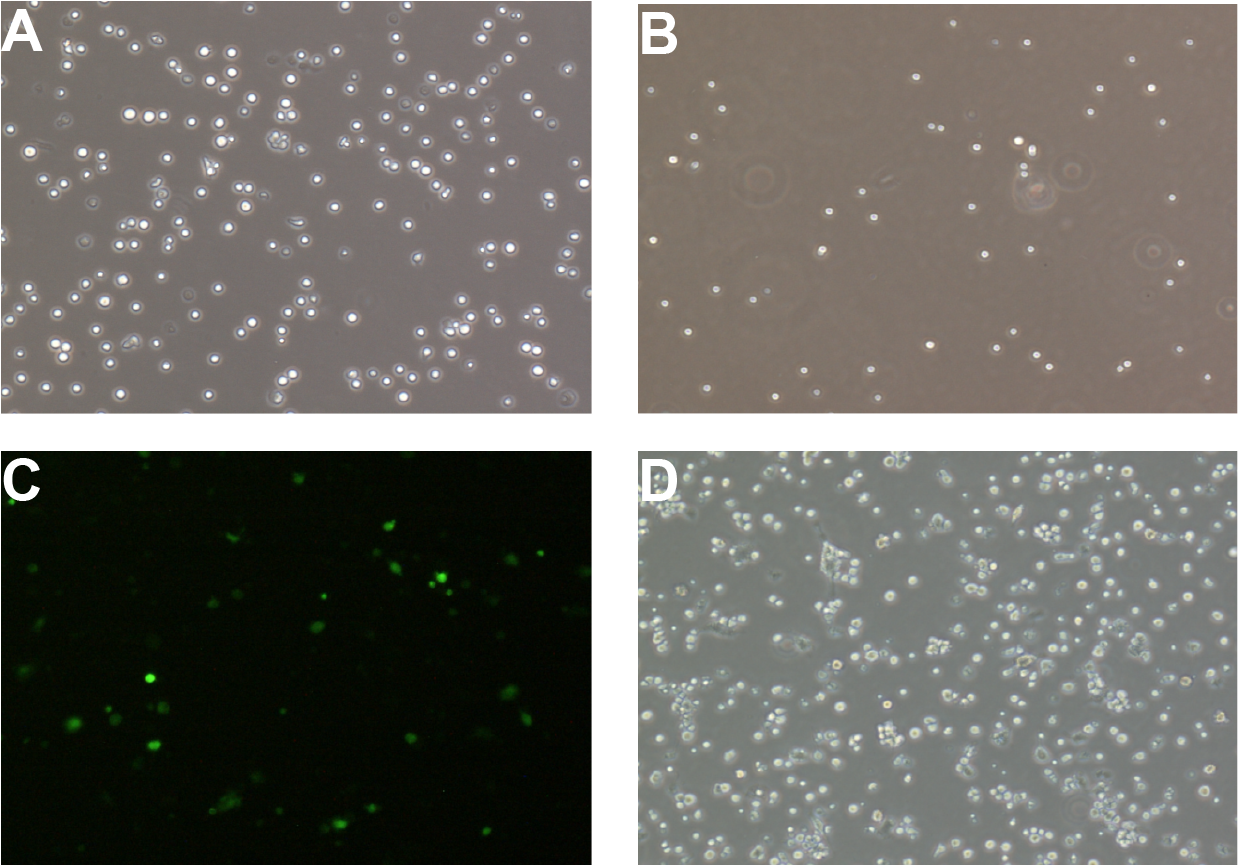

Supplement: Supplementary 2 — The activated and virus-transfected CD4+ T cells (A) the activated CD4+ T cells became bigger after ConA stimulation. (B) Cells that emit green fluorescence under blue excitation light are successfully transfected cells (×10 objective). [file 7783481.f2.png]
